# Type A Aortic Dissection Masquerading as an Inferior Myocardial Infarction

**DOI:** 10.1055/s-0041-1732396

**Published:** 2021-12-08

**Authors:** Azhar Hussain, Alessia Rossi, Alexander Smith, Ana Lopez-Marco, Amina Khalil, Neil Roberts

**Affiliations:** 1 Department of Cardiac Surgery, St. Bartholomew's Hospital, London, United Kingdom

**Keywords:** aortic dissection, myocardial infarction, percutaneous coronary intervention

## Abstract

Type A aortic dissection is a life-threatening condition with a wide range of clinical manifestations. Dissection can sometimes mimic an acute myocardial infarction due to similar presenting symptoms and initial clinical investigations. We report the case of a 52-year-old male who presented with an inferior ST-segment elevation myocardial infarction with two drug-eluting stents inserted as a stabilizing intervention prior to surgical repair of an acute aortic dissection.

## Introduction

Type A aortic dissection is a life-threatening condition with a wide range of clinical manifestations. 1 Aortic dissections can sometimes mimic an acute myocardial infarction due to similar presenting symptoms and initial clinical investigations. Misdiagnosis in such an instance can be catastrophic due to the immediate administration of thrombolytic and antiplatelet agents, which theoretically increase the mortality risk in this group. We report the case of a 52-year-old male who presented with an inferior ST-segment elevation myocardial infarction with two drug-eluting stents (DESs) inserted prior to the diagnosis of an acute aortic dissection.

## Case Presentation

A 52-year-old gentleman presented to hospital with central chest pain, diaphoresis, and collapse while at work. His past medical history was significant for hypertension, and he was a current smoker. Initial electrocardiogram (ECG) by the ambulance crew suggested inferior ST-segment elevation. He was urgently transferred to the cardiac catheterization laboratory for primary percutaneous coronary intervention (PCI). He was clinically in shock on arrival with a systolic blood pressure of 70 mm Hg and a heart rate of 40 in complete heart block. Initial contrast injection suggested severe ostial disease of the right coronary artery. A 3 mm × 30 mm XIENCE DES was inserted into the proximal vessel with hemodynamic improvement but with some persistent ST-elevation. The ostium appeared missed, so a second DES was deployed with both stents overlapping. ST-segments and hemodynamics subsequently improved with a heart rate of 120. No obvious aortic dissection was seen on the aortogram during the procedure.

**Fig. 1:**
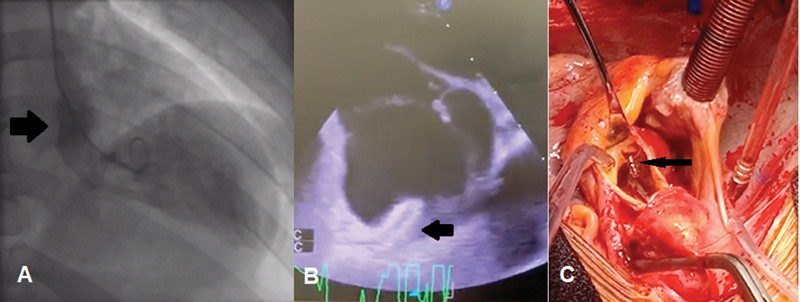
( **A** ) Left ventricular aortography showing the true lumen delineated by the dissection flap (arrow). ( **B** ) Transesophageal echocardiogram showing the right coronary artery (RCA) stent protruding into the aorta (arrow). ( **C** ) Intraoperative picture demonstrating RCA stent protruding from the ostium (arrow).

The patient was transferred to the ward for observation following the procedure. He became hypoxic requiring optiflow overnight, with episodes of hemoptysis. Clinical examination revealed signs of pulmonary edema and a diastolic murmur. Urgent review of the PCI images suggested possible aortic dissection ( Fig. 1 ). The echocardiography revealed free-flowing aortic regurgitation with a dissection flap visible in the ascending aorta which was confirmed on computed tomography aortogram.

The patient underwent an emergency aortic dissection repair with a mechanical aortic valve replacement, ascending aorta and hemiarch replacement, and coronary bypass graft to the right coronary artery (RCA). An intimal tear on the noncornary cusp at the level of the sinotubular junction was found. The DESs were found protruding into the aorta from the right coronary os and were removed ( Fig. 1 ). The patient was discharged 3 weeks later with almost complete recovery in follow-up at 6 months.

## Discussion

Acute aortic dissection can be a challenging diagnosis to make in an emergency setting. ECGs often show some degree of nonspecific ST-segment or T-wave changes, but changes suggestive of an acute myocardial infarction in aortic dissection are rare. 2 Misdiagnosis can have potential deleterious consequences as a result of antiplatelet and thrombolytic agents given prior to surgical repair. The involvement of the RCA is well documented and attributed to dissection flaps more commonly originating in the anterior aspect of the ascending aorta above the sinuses of Valsalva. 3

Although misdiagnosis occurred in our case, it is debatable that early administration of a DES and antiplatelet agents allowed adequate reperfusion for the myocardium to recover from the initial shock and permit successful surgical repair. Hemodynamics, ST-segment changes, and complete heart block all resolved once the second DES was deployed, with a view to discharging the patient in the coming days. Had the dissection been visualized prior to DES deployment, it is likely that the patient would have been referred directly to the operating room without sufficient protection of the right coronary territory. In this case, PCI acted as a bridge to a successful repair of an acute aortic dissection complicated by an acute RCA territory infarct. The requirement of two overlapping stents was likely secondary to the space created by the dissection flap, displacing the true ostium. Intraoperatively, the DES could be seen protruding into the aorta and was removed. A coronary bypass graft was placed to the mid-RCA to ensure adequate flow which may have been compromised at the ostium due to the manipulation of the stents.

This case highlights the importance of being vigilant in a patient who presented with an acute myocardial infarction. The diagnosis of aortic dissection can easily be missed in such cases. Thorough clinical examination is needed, as well as careful interpretation of available imaging studies. Although this patient was misdiagnosed, percutaneous coronary intervention to the RCA most likely kept the patient clinically stable until definitive surgical management for his aortic dissection.
